# Anesthesiologists in China: a nationwide cross-sectional survey of working conditions, clinical practice, and career challenges

**DOI:** 10.3389/fpubh.2026.1846157

**Published:** 2026-07-03

**Authors:** Yixun Lu, Changsheng Zhang, Weihua Wang, Xuecai Lv, Huikai Yang, Yulong Ma, Jiangbei Cao, Yuguang Huang, Weidong Mi

**Affiliations:** 1 Department of Anesthesiology, The First Medical Center of Chinese PLA General Hospital, Beijing, China; 2 Department of the Prevention and Control of Noncommunicable Chronic Diseases, Shaanxi Provincial Center for Disease Control and Prevention, Xi'an, Shaanxi, China; 3 School of Medicine, Nankai University, Tianjin, China; 4 Department of Anesthesiology, Peking Union Medical College Hospital, Beijing, China; 5 National Clinical Research Center for Geriatric Diseases, Chinese PLA General Hospital, Beijing, China

**Keywords:** anesthesiologists, health service, nationwide survey, policy-making, working conditions

## Abstract

**Background:**

Anesthesiologists play a critical role in healthcare system, yet the overall working conditions of anesthesiologists in China remain unclear. This study aimed to conduct a comprehensive national assessment of this workforce.

**Methods:**

An open nationwide, cross-sectional, non-probability sampling survey focusing on working conditions, clinical practice and career challenges of anesthesiologists across China was conducted by Chinese Society of Anesthesiology from June 2022 to February 2023. The questionnaire was distributed via WeChat online platform to anesthesiology practitioners across all healthcare tiers and diverse regions of mainland China. Data were analyzed and visualized using R software. Logistic regression analysis was conducted to identify factors associated with resignation intention.

**Results:**

A total of 30,255 participants from 5,808 medical institutions were included in the analysis, with females accounting for 47.28%. Overall, 16.29% of participants reported resignation intention. Regression analyses identified significant associations between resignation intention and service years, weekly work hours, daily anesthesia caseload, annual income, sleep quality, smoking, alcohol, and exercise frequency. A majority (62.72%) participants reported weekly meaningful work accomplishment, while 50.4% reported weekly work overload, and 30.1% experienced weekly work-induced exhaustion. In addition, 82.12% of participants never treated patients with indifference. Overall, 56.27% reported job satisfaction. Chinese anesthesiologists exhibited high self-reported clinical quality and efficacy, with low rate of adverse events (2.99%). Medical disputes (7.44%) and physical violence (4.91%) were rare, while 35.32% reported experiencing verbal violence from patients. The primary barrier to professional title promotion was paper publication (62.81%).

**Conclusion:**

Despite demonstrating high clinical quality and professional fulfillment, Chinese anesthesiologists face substantial challenges including heavy workloads, occupational burnout, and career advancement barriers. These findings provide critical insights for targeted interventions and inform evidence-based policy-making to ensure a sustainable, high-quality anesthesia workforce in China.

## 1 Introduction

Anesthesiologists serve as indispensable components within the healthcare system, providing essential services spanning diagnostic procedures, perioperative management, critical care, and pain medicine. Nevertheless, global evidence shows persistent shortages and marked geographic maldistribution of the anesthesia workforce, particularly in low- and middle-income settings (1, 2). The World Federation of Societies of Anesthesiologists has highlighted the need to strengthen physician anesthesia-provider density and workforce planning as part of global surgical and public-health capacity (3). At the same time, anesthesiologists worldwide face fatigue, burnout, and career-sustainability challenges, all of which may affect workforce retention and quality of care. This challenge might be particularly evident in Chinese context. (4–6). The anesthesia workforce routinely experiences systemic pressures including critical shortages in physician supply, excessive clinical workloads, and significant occupational stress, culminating in high prevalence rates of anxiety, depressive symptoms, and professional burnout (6, 7). Notably, China's anesthesiologist density (0.4 per 10,000 population) lags markedly behind those documented in developed nations (2.4–3.0 per 10,000) (8, 9). Regional investigations, such as one conducted across the Beijing-Tianjin-Hebei region, reported burnout affecting 69% of surveyed anesthesiologists (10). Most recently, findings from another survey in 2023 revealed burnout persisted post-COVID at 52.7% nationally (11). Compounding these occupational issues are substantial exposure to workplace hazards (12, 13), increasingly strained doctor-patient dynamics, perceived misalignment between workload intensity and professional remuneration, and inadequate public recognition (14) of their pivotal contributions to patient safety and surgical outcomes (15). Collectively, this occupational milieu compromises both clinical quality and long-term sustainability of the anesthesia workforce.

In China, the specialty of anesthesiology has expanded rapidly. It is estimated that there are approximately 105,732 anesthesiologists in China (16), yet nationally detailed evidence on anesthesiologists' work conditions remains incomplete. Existing Chinese studies have provided valuable insights but have often been limited by regional scope, modest sample size, or focus on selected hospital types (10, 17, 18). Specifically, practice circumstances and challenges encountered within primary and secondary healthcare settings remain poorly characterized (19). Moreover, existing questionnaires often lack comprehensive scope, failing to adequately capture the interplay of complex factors (20). Consequently, considerable uncertainty persists regarding the true working conditions across China.

To address these evidence gaps, the Chinese Society of Anesthesiology (CSA), a national specialty branch of the Chinese Medical Association established in 1979, organized a large-scale nationwide survey between June 2022 to February 2023. This investigation aimed to comprehensively assess working conditions, self-reported clinical-practice experience, job satisfaction, occupational risks, and career-development challenges of anesthetic providers (for the sake of simplicity, we use the term anesthesiologist). The findings are intended to contributes to the literature and support evidence-informed workforce planning, occupational-health interventions, and policy development for sustainable anesthesia care in China.

## 2 Methods

### 2.1 Study design and participants

This study adopted a nationwide, cross-sectional open survey design, formally orchestrated and administered by the Chinese Society of Anesthesiology (CSA) via WeChat platform from June 2022 to February 2023. This study employed open voluntary non-probability sampling and potential selection bias might be introduced. Relying on the CSA organizational infrastructure, extensive pre-investigation dissemination and mobilization were implemented to optimize participation and maximize national coverage. Target participants comprised anesthesiology practitioners working in mainland Chinese medical institutions. Participants were recruited from all tiers of healthcare facilities and diverse geographic regions to ensure broad national coverage. Participation was strictly voluntary. Electronic informed consent was obtained from all participants. It was permitted to review or change the answers before submitting the questionnaire. After the respondents submit the questionnaires, the answers cannot be changed anymore. Personal information was anonymized and protected.

As a methodological benchmark, the minimum sample size for this survey was estimated using the standard formula for cross-sectional studies: *n* = Z^2^ × P (1–P)/d^2^, where *Z* = 1.96 (95% confidence level), *P* = 0.5 (maximal variance to ensure sufficient sample), *d* = 0.03 (margin of error). The calculated minimum sample size was approximately 1,067. Considering potential non-response, missing data, and regional diversity across mainland China, we aimed to recruit a much larger sample to improve precision and maximize national coverage.

### 2.2 Questionnaire development and validation

The survey questionnaire was developed to comprehensively evaluate the multidimensional working conditions of anesthesiology workforce in China. Questionnaire design was based on literature review (6, 7, 10, 21, 22), expert consultation, and pilot testing. A multidisciplinary expert panel comprising senior anesthesiologists and health services researchers critically evaluated item relevance, comprehensiveness, and appropriateness. A pilot test involving 259 practitioners was conducted to evaluated item comprehensibility, response feasibility, and domain coverage. Psychometric validation was performed to evaluate reliability and validity. Cronbach's α coefficient was 0.872 and Guttman split-half coefficient was 0.826, both above 0.7, indicating good internal consistency reliability. Construct validity was examined using exploratory factor analysis. The KMO value was 0.853 (>0.8), and Bartlett's test of sphericity was significant (*P* < 0.001), supporting the appropriateness of factor analysis. Principal component analysis extracted a clear theoretical factor structure consistent with the study domains, and all factor loadings were above 0.4, indicating satisfactory construct validity. The questionnaire was subsequently optimized and refined before final release. The questionnaire specifically addressed four key dimensions relevant to the Chinese context: 1) Demographic and professional characteristics; 2) Work feelings and experiences, such as occupational burnout (modified Maslach Burnout Inventory) and stress coping strategies; 3) Clinical practice quality, such as clinical medical safety and adverse event reporting; and 4) Career development challenges and wellbeing. For the complete questionnaire, please refer to Supplementary File 1.

### 2.3 Data collection and quality control

Data were collected anonymously via an electronic questionnaire administered through WeChat, utilizing the REDCap^®^ platform (Vanderbilt University, Nashville, Tennessee, USA, Version 12.4.30, http://redcap.bjmu.edu.cn/), a secure and flexible data collection platform with built-in logical verification and mandatory response for critical variables to reduce errors. The CSA distributed the survey link to anesthesiology departments and practitioners nationwide through its established national network. Because the survey was distributed through a professional network and no closed invitation denominator was available, a conventional response rate could not be calculated. Instead, we report the valid submission rate and national workforce coverage.

**Figure 1:**
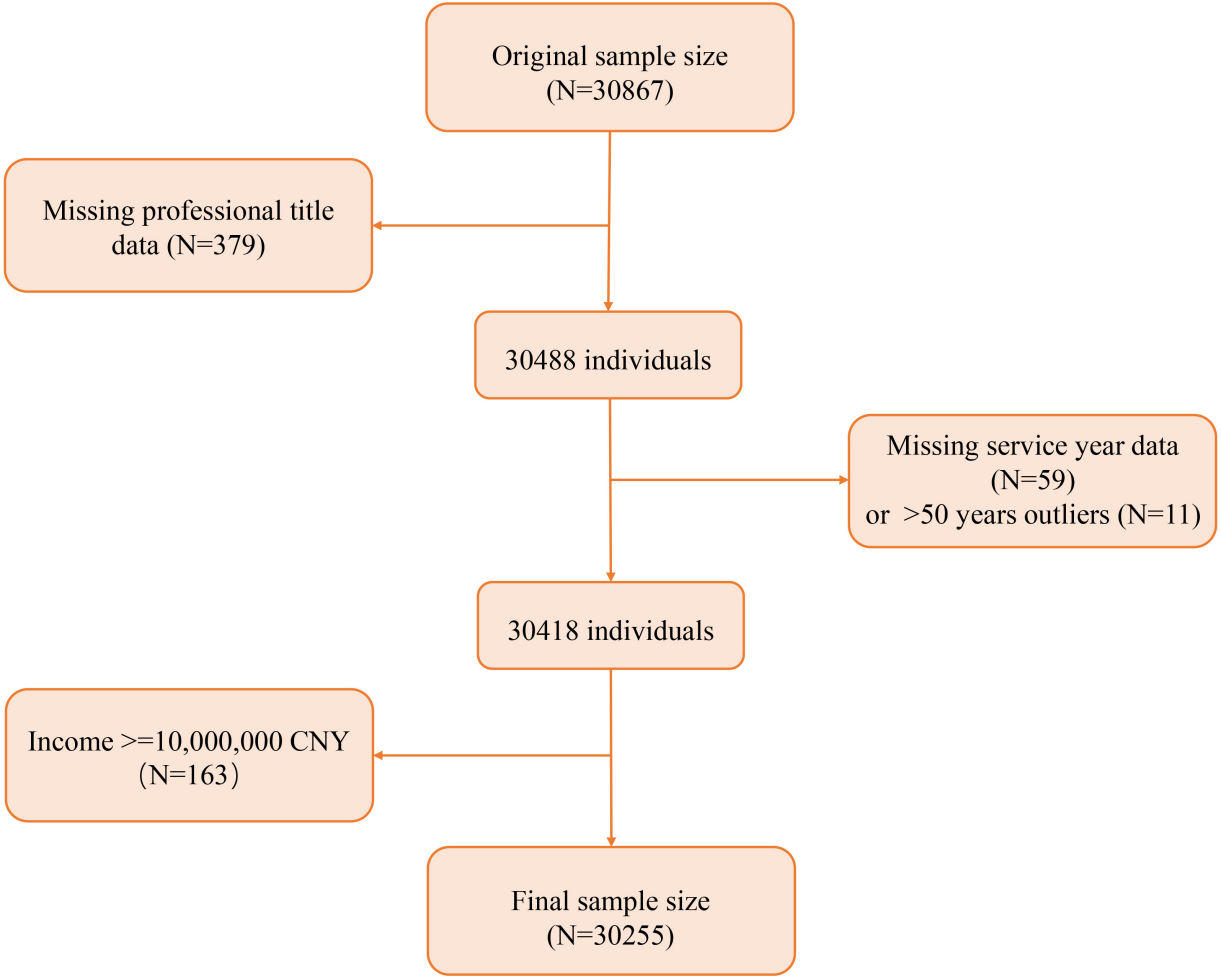
The flowchart of participants selection. CNY, China Yuan.

To ensure data reliability, multiphase quality control measures include: 1) Standardized training and certification of provincial CSA coordinators before the investigation; 2) Real-time monitoring, logical verification and identity deduplication during the investigation; 3) manual data verification after the investigation. After excluding submissions with missing data (*n* = 438, proportion = 1.42%) and outlier values (*n* = 174, proportion = 0.56%), systematic curation of the initial 30,867 responses yielded 30,255 analyzable cases and a valid submission rate of 98.02%. The participant selection process is formalized in Figure 1.

### 2.4 Statistical analysis

Data analysis was performed using EmpowerStats (Version 6.0, www. empowerstats.com). Descriptive statistics were employed to summarize participant characteristics and responses to questionnaire items. Continuous variables are presented as mean ± standard deviation (SD) or the median with interquartile range (IQR) based on whether they conform to the normal distribution, while categorical and ordinal variables are described as frequencies (*n*) and percentages (%).

Univariate analyses were conducted to examine associations between various baseline characters and resignation intention, the primary outcome. For continuous variables, the Wald test *P*-value was calculated. For categorical variables, Chi-square or Fisher's exact tests were used as appropriate. For variables potentially associated with resignation idea, multivariable logistic regression was carried out, calculating Odds Ratios (OR) and 95% Confidence Intervals (CI). Variables were entered into the multivariable logistic regression model based on both univariate statistical significance (*P* < 0.05) and clinical relevance (regardless of the *P* value of univariate analysis). Candidate variables included demographic characteristics, work-related factors (weekly work hours, daily anesthesia cases, service years), income, sleep quality, lifestyle factors (smoking, alcohol consumption, exercise), and job-related conditions. *P* values were adjusted with Bonferroni methods.

Restricted Cubic Spline (RCS) analysis was utilized to model potential non-linear relationships between service years, annual income and the possibility of resignation idea, with threshold effect analysis identifying inflection point. R software (Version 4.2.1) “ggplot2” package was used for visualization. Statistical significance was defined as *P* < 0.05.

## 3 Results

### 3.1 Baseline characters of participants, univariate and multivariable logistic regression analysis

**Figure 2:**
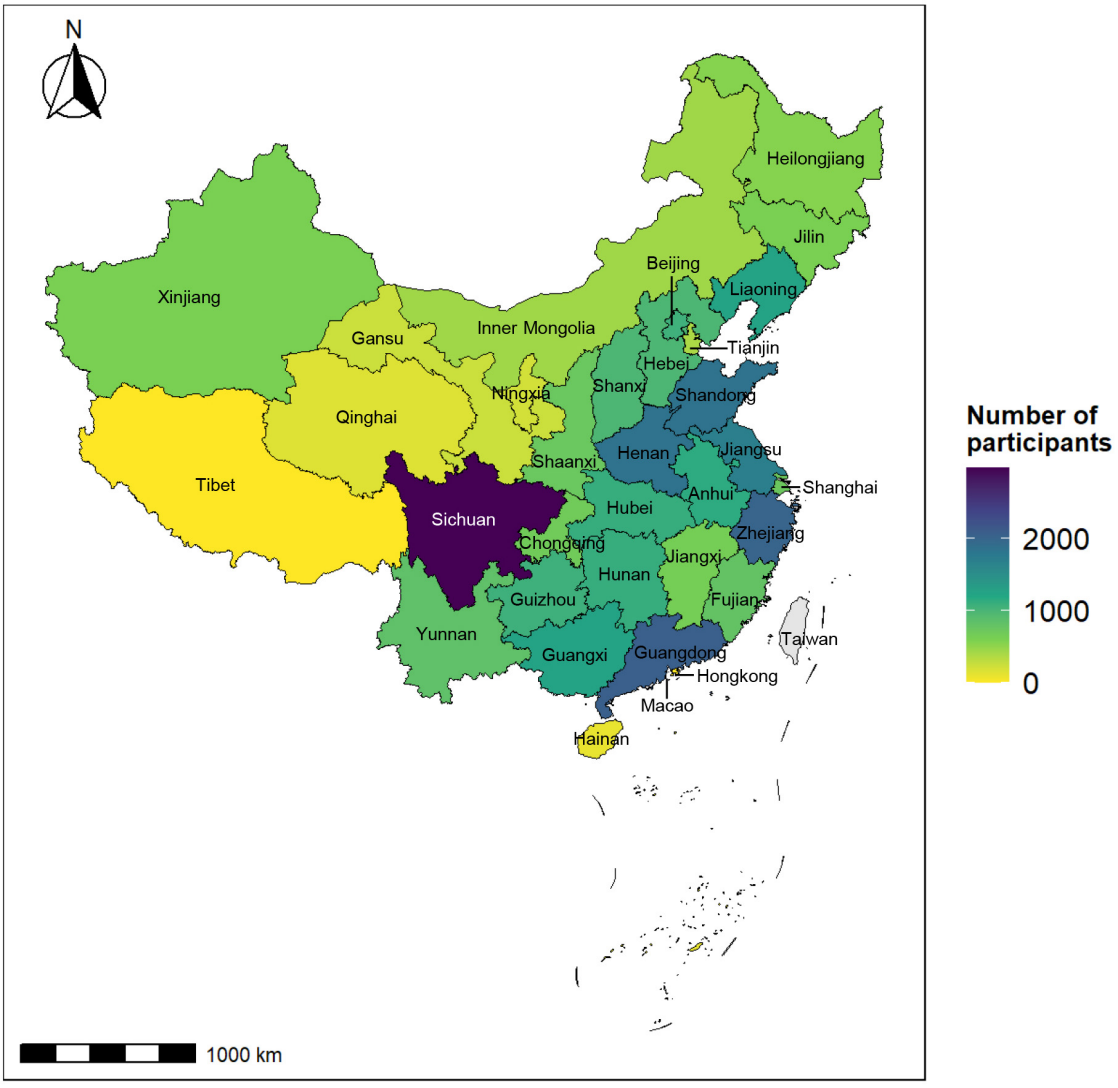
Map of participants distribution. Across mainland China, Sichuan (2,985), Guangdong (2,073), Zhejiang (2,017), Shandong (1,855), and Henan (1,850) provinces contributed the largest number of participants.

A total of 30,255 anesthesiologists from 5,808 medical institutions across China were included in the analysis. The median age of Participants was 36 (IQR 31–42) years, and 47.28% (*n* = 14,304) were female. Figure 2 and Supplementary Figure S1 presents the regional distribution of participants, with the highest contributions from Sichuan, Guangdong, Zhejiang, Shandong, and Henan provinces. Demographic and professional characteristics of participants are detailed in Table 1.

**Table 1:** Demographic and professional characteristics of participating anesthesiologists (*N* = 30,255).

Characters	Statistics
Gender	
Male	15,951 (52.72%)
Female	14,304 (47.28%)
**Age**	36 (IQR 31–42)
Hospital level	
Grade 1	568 (1.88%)
Grade 2	9,263 (30.62%)
Grade 3	20,168 (66.66%)
Ungraded and others	256 (0.84%)
Hospital type	
Private hospital	2,884 (9.53%)
Public TCM hospital	1,520 (5.02%)
Public specialized hospital	3,717 (12.29%)
Public general hospital	22,009 (72.75%)
Other	125 (0.41%)
Identity	
Permanent staff	21,771 (71.96%)
Contract staff	7,876 (26.03%)
Rotational or trainees	337 (1.11%)
15.6-7.2,-1.3242ptOthers	271 (0.90%)
Profession	
Certified doctor	28,113 (92.92%)
Physician assistant	1,057 (3.49%)
Nurse	789 (2.61%)
Technician	103 (0.34%)
Others	193 (0.64%)
Major	
Anesthesiology	22,007 (72.74%)
Clinical Medicine	7,319 (24.19%)
Nursing	816 (2.70%)
Others	113 (0.37%)
Education	
Doctoral and master degree	7,346 (24.28%)
Bachelor degree and below	22,909 (75.72%)
Profession title	
Junior	9,762 (32.27%)
Intermediate	13,614 (45.00%)
Associate senior	5,234 (17.30%)
Senior	1,293 (4.27%)
Other	352 (1.16%)
**Service years**	10 (IQR 5–16)
Week work hours	
< 40 h	1,886 (6.23%)
40–50 h	11,965 (39.55%)
50–60 h	10,307 (34.07%)
60–70 h	3,921 (12.96%)
70–80 h	1,303 (4.31%)
>80 h	873 (2.89%)
Day anesthesia cases	
1 case	2,196 (7.26%)
2–3 cases	11,674 (38.59%)
4–5 cases	11,334 (37.46%)
6–7 cases	3,117 (10.30%)
8–10 cases	1,067 (3.53%)
>10 cases	867 (2.87%)
On-call duty	
No	7,220 (23.86%)
Yes	23,035 (76.14%)
Period of on duty (among respondents with on-duty shifts, *n* = 22,900)	
< =3 days	3,573 (15.60%)
4–10 days	17,580 (76.77%)
>10 days	1,747 (7.63%)
**Income per year**	11 (IQR 8–16)
Marital status	
Unmarried	5,218 (17.25%)
Married without children	2,717 (8.98%)
Married with minor children	18,989 (62.76%)
Married with adult children	3,124 (10.33%)
Others	207 (0.68%)
Sleep quality	
Very good	3,515 (11.62%)
Good	8,034 (26.55%)
Average	12,518 (41.37%)
Poor	4,953 (16.37%)
Very poor	1,235 (4.08%)
Smoking status	
No	25,182 (83.23%)
Former smoker, but has quit	1,870 (6.18%)
Yes, still smoking currently	3,203 (10.59%)
Alcohol status	
Never	16,127 (53.30%)
Once or less per month	9,137 (30.20%)
2–4 times per month	3,730 (12.33%)
2–3 times per week	1,002 (3.31%)
4 times or more per week	259 (0.86%)
Exercise frequency	
No exercise	14,185 (46.88%)
Once a week	8,209 (27.13%)
2–3 times a week	5,720 (18.91%)
4–5 times a week	1,091 (3.61%)
Almost everyday	1,050 (3.47%)

IQR, inter-quartile range.

Overall, 16.29% (*n* = 4,929) of participants reported resignation intention in the past month. Univariate analysis identified several demographic and occupational factors significantly associated with resignation intention among Chinese anesthesiologists. These included gender, age, hospital level, hospital type, employment identity, profession, major, service years, weekly working hours, daily anesthesia cases, on-call duty status and frequency, annual income, marital status, sleep quality, smoking status, alcohol consumption, and exercise frequency (all *P* < 0.05). Detailed results of the univariate analysis are presented in Supplementary Table S1.

Specifically, univariate analysis revealed that female anesthesiologists had a lower likelihood of reporting resignation intention compared with their male counterparts (OR = 0.91, 95% CI: 0.86–0.97). Compared with anesthesiologists in higher-level hospitals, those working in primary hospitals had a significantly lower likelihood of reporting resignation intention (OR = 0.48, 95% CI: 0.24–0.99). Anesthesiologists in public general hospitals were more likely to consider resignation than their counterparts in private hospitals (OR = 1.15, 95% CI: 1.04–1.29). Contract staff exhibited a higher likelihood of resignation intention compared with permanent staff (OR = 1.43, 95% CI: 1.34–1.53). Smoking and alcohol consumption were positively associated with resignation intention, whereas regular exercise demonstrated a protective effect. Individuals who engaged in physical activity 2–3 times per week showed remarkable protective association (OR = 0.51, 95% CI: 0.46–0.55). Regarding marital status, married anesthesiologists with adult children had the lowest odds of considering resignation (OR = 0.49, 95% CI: 0.43–0.57).

**Figure 3:**
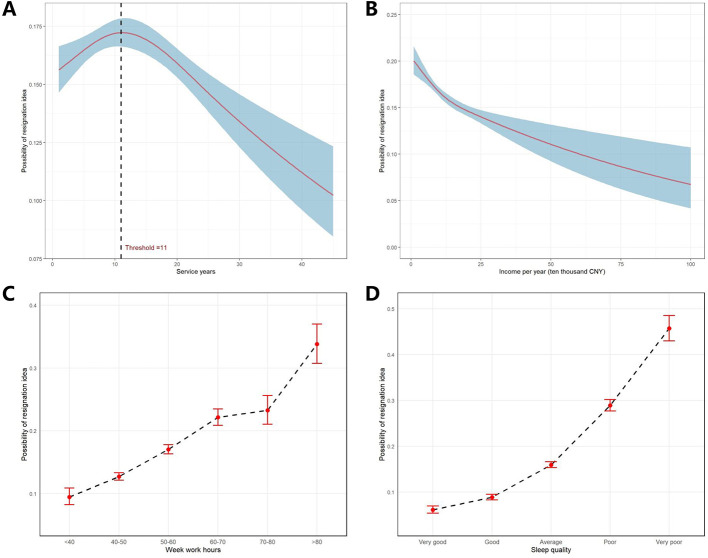
**A** and **B** show the relationship between service years, annual income and possibility of resignation idea. The red solid line represents the smooth fitting curve of annual income and resignation risk, and the blue shaded area represents the 95% confidence interval. Panels **C** and **D** show the relationship between week work hours, sleep quality and likelihood of resignation. The black dashed line represents possibility of resignation idea, and the red solid line represents the 95% confidence interval.

Restricted cubic spline analysis demonstrated a non-linear association between service years and resignation intention, with an inverted U-shaped relationship (the shaded area represented the 95% CI) peaking at approximately 11 years (95%CI: 5.77–16.23) of service (Figure 3A). Annual income was inversely associated (the shaded area represented the 95% CI) with the likelihood of resignation intention (Figure 3B). In contrast, longer weekly working hours showed a positive relationship with resignation intention, as illustrated by piecewise linear regression analysis (Figure 3C). A similar pattern was observed for daily anesthesia cases, with higher caseloads associated with increased odds of considering resignation. Furthermore, participants who reported poor or very poor sleep quality were more likely to contemplate resignation than those with good sleep quality (Figure 3D).

Multivariable logistic regression analysis identified service years (OR = 1.03, 95% CI: 1.02–1.05), weekly working hours exceeding 60 h, and alcohol consumption as prominent independent risk factors (all *P* < 0.001) for resignation intention. In contrast, higher annual income (OR = 0.98, 95% CI: 0.97–0.99), being married with adult children (OR = 0.52, 95% CI: 0.39–0.69), good sleep quality and moderate exercise emerged as crucial independent protective factors (all *P* < 0.001) (Supplementary Table S2).

### 3.2 Work feelings and occupational experiences of anesthesiologists in their job

**Figure 4:**
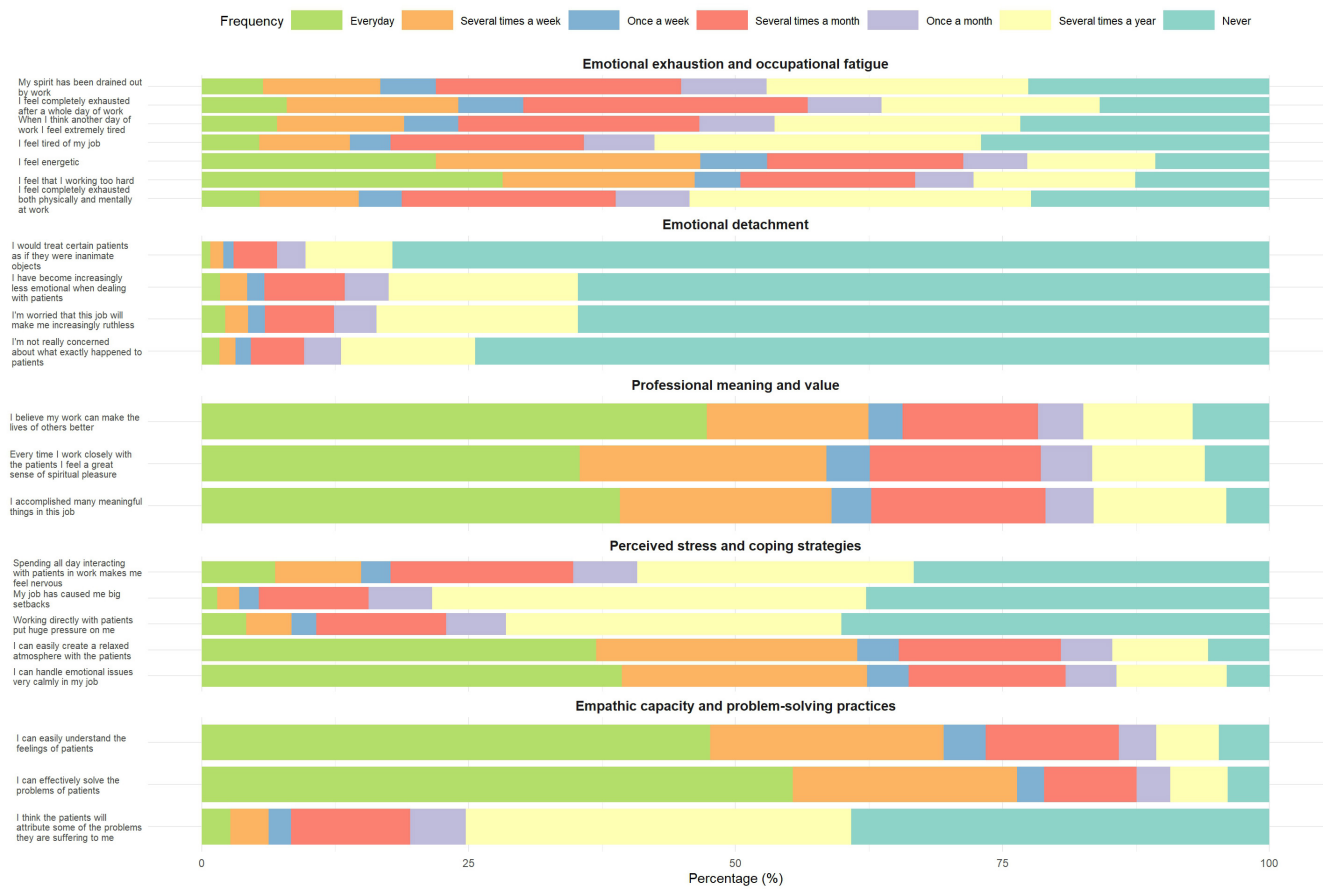
The work feelings and occupational experiences of Chinese anesthesiologists. Five sub-themes were evaluated: Emotional exhaustion and occupational fatigue, Emotional detachment, Perception of professional meaning and value, Perceived stress and coping strategies, Empathic capacity and problem-solving practices.

Questionnaire results of the work feelings and occupational experiences revealed significant levels of occupational burnout and emotional exhaustion (Figure 4). The core results and full results of work feelings and occupational experiences of anesthesiologists were presented in Table 2 and Supplementary Table S3, respectively.

**Table 2:** Self-reported work feelings and occupational experiences of anesthesiologists in China (*N* = 30,255).

Questionnaire items	Statistics [N (%)]
My spirit has been drained out by work	
Never	6,829 (22.57%)
Several times a year	7,419 (24.52%)
Once a month	2,423 (8.01%)
Several times a month	6,942 (22.94%)
Once a week	1,574 (5.20%)
Several times a week	3,327 (11.00%)
15.6-7.2,-1.3242ptEveryday	1,741 (5.75%)
I feel completely exhausted after a whole day of work	
Never	4,813 (15.91%)
Several times a year	6,173 (20.40%)
Once a month	2,091 (6.91%)
Several times a month	8,064 (26.65%)
Once a week	1,842 (6.09%)
Several times a week	4,864 (16.08%)
15.6-7.2,-1.3242ptEveryday	2,408 (7.96%)
I can effectively solve the problems of patients	
Never	1,188 (3.93%)
Several times a year	1,626 (5.37%)
Once a month	955 (3.16%)
Several times a month	2612 (8.63%)
Once a week	773 (2.55%)
Several times a week	6,350 (20.99%)
Everyday	16,751 (55.37%)
I believe my work can make the lives of others better	
Never	2,168 (7.17%)
Several times a year	3,108 (10.27%)
Once a month	1,277 (4.22%)
Several times a month	3,845 (12.71%)
Once a week	958 (3.17%)
Several times a week	4,588 (15.16%)
Everyday	14,311 (47.30%)
I'm worried that this job will make me increasingly ruthless	
Never	19,596 (64.77%)
Several times a year	5,701 (18.84%)
Once a month	1,212 (4.01%)
Several times a month	1,953 (6.46%)
Once a week	475 (1.57%)
Several times a week	651 (2.15%)
Everyday	667 (2.20%)
My job has caused me big setbacks	
Never	11,433 (37.79%)
Several times a year	12,287 (40.61%)
Once a month	1,808 (5.98%)
Several times a month	3,105 (10.26%)
Once a week	561 (1.85%)
Several times a week	616 (2.04%)
15.6-7.2,-1.3242ptEveryday	445 (1.47%)
In this job, I accomplished many meaningful things	
Never	1,223 (4.04%)
Several times a year	3,761 (12.43%)
Once a month	1,366 (4.51%)
Several times a month	4,930 (16.29%)
Once a week	1,124 (3.72%)
Several times a week	6,003 (19.84%)
Everyday	11,848 (39.16%)
At work, I feel completely exhausted both physically and mentally	
Never	6,759 (22.34%)
Several times a year	9,668 (31.96%)
Once a month	2,092 (6.91%)
Several times a month	6,068 (20.06%)
Once a week	1,211 (4.00%)
Several times a week	2,809 (9.28%)
Everyday	1,648 (5.45%)

#### 3.2.1 Emotional exhaustion and occupational fatigue

Regarding emotional exhaustion, 21.95% of respondents reported experiencing work-induced spiritual drain at least once per week, including 5.75% who reported this experience daily. Similarly, 30.13% experienced complete exhaustion after work ≥1 time/week, with 7.96% reporting it daily. Nearly a quarter of participants (24.03%) felt extreme tiredness facing a new workday at least weekly, including 7.07% daily. Over a third (35.79%) felt tired of their job at least several times per month, including 5.41% reporting this feeling daily. While 21.95% reported feeling energetic daily, 28.65% reported experiencing this feeling only monthly, several times a year, or never. However, a significant proportion (72.31%) reported feeling overworked at least once per month, and 50.44% reported this at least once per week, including 28.19% daily. Almost one in five participants (18.73%) reported experiencing complete physical and mental exhaustion at least once per week, with 5.45% experiencing this daily.

#### 3.2.2 Emotional detachment

Treating patients in a depersonalized manner was uncommon, with 82.1% of respondents reporting that they “never” did so. However, 17.5% reported feeling less emotionally connected to or empathetic toward patients at least once a month, including 5.9% who experienced this at least weekly. Concern about becoming increasingly ruthless was reported as “never” experienced by 64.8% of participants, whereas 16.4% reported experiencing this worry at least monthly. A lack of concern for patients was relatively infrequent, with 74.4% indicating they “never” felt this way and only 4.6% reporting indifference at least weekly.

#### 3.2.3 Perception of professional meaning and value

A majority of participants reported believing their work improved others' lives at least weekly (65.63%), including 47.30% daily. Similarly, 62.72% reported accomplishing meaningful work at least weekly, with 39.16% reporting this daily. Spiritual fulfillment derived from patient care was also frequently reported, with 62.58% reporting it at least weekly and 35.42% daily.

#### 3.2.4 Perceived stress and coping strategies

A significant proportion of participants (40.78%) reported experiencing nervousness from workplace social interactions least monthly, with 17.69% reporting this at least weekly. Pressure from direct patient care was reported by 28.41% at least monthly, including 10.72% at least weekly. The majority (65.31%) reported easily creating a relaxed physician-patient atmosphere at least weekly, with 36.93% doing so daily. Similarly, 66.21% reported managing emotional issues effectively at least weekly, including 39.35% daily.

#### 3.2.5 Empathic capacity and problem-solving practices

The majority of participants reported easily understanding patients' feelings frequently (73.45% at least weekly, including 47.62% daily). A high proportion of participants also reported confidence in effectively solving patients' problems (78.91% at least weekly, including 55.37% “Everyday”). Concern that patients attributed their problems to the anesthesiologist was uncommon, with 39.18% reporting this “Never” and 36.09% reporting it “Several times a year”.

### 3.3 Clinical practice quality and career development of anesthesiologists

Detailed findings regarding clinical practice quality, occupational challenges, and career development trajectories are presented in Table 3.

**Table 3:** Clinical practice quality, occupational risks, and career development indicators of Chinese anesthesiologists (*N* = 30,255).

Questionnaire items	Statistics
In general, are you satisfied with your job?	
Extremely dissatisfied	580 (1.92%)
Dissatisfied	1,274 (4.21%)
Average satisfaction	11,377 (37.60%)
Satisfied	13,025 (43.05%)
30-7.2,-1.3242ptVery satisfied	3,999 (13.22%)
The frequency at which you encounter challenging cases was?	
Few	4,244 (14.03%)
Once a month	15,767 (52.11%)
Once a week	6,461 (21.36%)
Once every 2–3 days	2,996 (9.90%)
Almost everyday	787 (2.60%)
In the past 3 months, the average communication time you spent with each patient and their family members was?	
Few	1,408 (4.65%)
1–3 min	4,956 (16.38%)
4–5 min	11,023 (36.43%)
6–10 min	7,858 (25.97%)
>10 min	5,010 (16.56%)
In the past 3 months, have you made any mistakes that caused harm to patients?	
No	29,348 (97.01%)
Yes	906 (2.99%)
**How many days of paid vacation do you have as a regular entitlement each year?**	5.0 (4.0–10.0)
The biggest challenge you are facing in terms of professional title promotion is?	
No difficulties	1,783 (5.89%)
Clinical competence	1,657 (5.48%)
Professional title examination	4,269 (14.11%)
Paper publication	19,004 (62.81%)
Competition among colleagues	2,224 (7.35%)
Other difficulties	1,318 (4.36%)
In the past year, have you ever experienced medical disputes?	
No	28,003 (92.56%)
Yes	2,252 (7.44%)
In the past year, the frequency of verbal violence that you have experienced from the patients was?	
None	19,569 (64.68%)
Less than once per month	8,391 (27.73%)
Once per month	1,047 (3.46%)
2–3 times per month	967 (3.20%)
Once per week	141 (0.47%)
2–5 times per week	73 (0.24%)
Almost everyday	67 (0.22%)
In the past year, the frequency of physical violence that you have experienced from the patients was?	
None	28,768 (95.09%)
Less than once per month	1,140 (3.77%)
Once per month	141 (0.47%)
2–3 times per month	112 (0.37%)
Once per week	51 (0.17%)
2–5 times per week	15 (0.05%)
Almost everyday	28 (0.09%)
In the past month, have you ever had the idea of resignation?	
No	25,326 (83.71%)
Yes	4,929 (16.29%)
In the past month, your actual sleep duration each night was?	
< 6 h	13,792 (45.59%)
6–7 h	13,312 (44.00%)
>7 h	3,151 (10.41%)

#### 3.3.1 Job satisfaction and workload

Overall, more than half of the respondents (56.3%) reported being satisfied or very satisfied with their jobs. In terms of encountering challenging cases, the majority (85.97%) reported experiencing challenging cases once a month or more frequently, while 14.03% reported encountering them infrequently. The most common communication duration with each patient/family was 4–5 min (36.43%), followed by 6–10 min (25.97%).

#### 3.3.2 Self-reported safety indicators and adverse events

The vast majority (76.75%) reported no instances of leaving patients unattended in the operating room for >5 min under general anesthesia in the past 3 months. Operational errors, such as deep vein puncture or intraspinal anesthesia, were reported by 25.81%, with 14.33% reporting errors only once, 10.07% occurring once or twice a month, and 1.41% reporting weekly or more frequent errors. Medication errors were infrequent, with 95.25% reporting no errors. Clinical judgment errors were reported by 15.22%, and 2.99% reported making mistakes that caused harm to patients in the past 3 months. The remainder reported no such incidents.

#### 3.3.3 Occupational risks and violence exposure

Medical disputes were reported by 7.44% of participants in the past year. Experiencing verbal violence from patients was reported by 35.32%, while physical violence was rare (4.91%). The majority of participants reported no incidents of medical disputes (92.56%), verbal violence (64.68%), or physical violence (95.09%).

#### 3.3.4 Career development and wellbeing

The reported median annual paid vacation was 5.0 (IQR 4.0–10.0) days. The most significant challenge for title promotion was paper publication (62.81%). Within the past month, 16.29% of participants had considered resignation. Regarding career choice, 30.32% were very likely and 22.65% were likely to choose their current career again. However, 28.49% were uncertain, 10.66% were less likely, and 7.88% stated it was impossible. The most common nightly sleep duration was 6–7 h (44.00%), followed by 5–6 h (38.24%). Additionally, 9.43% reported using sleep aid medications in the past month.

## 4 Discussion

**Figure 5:**
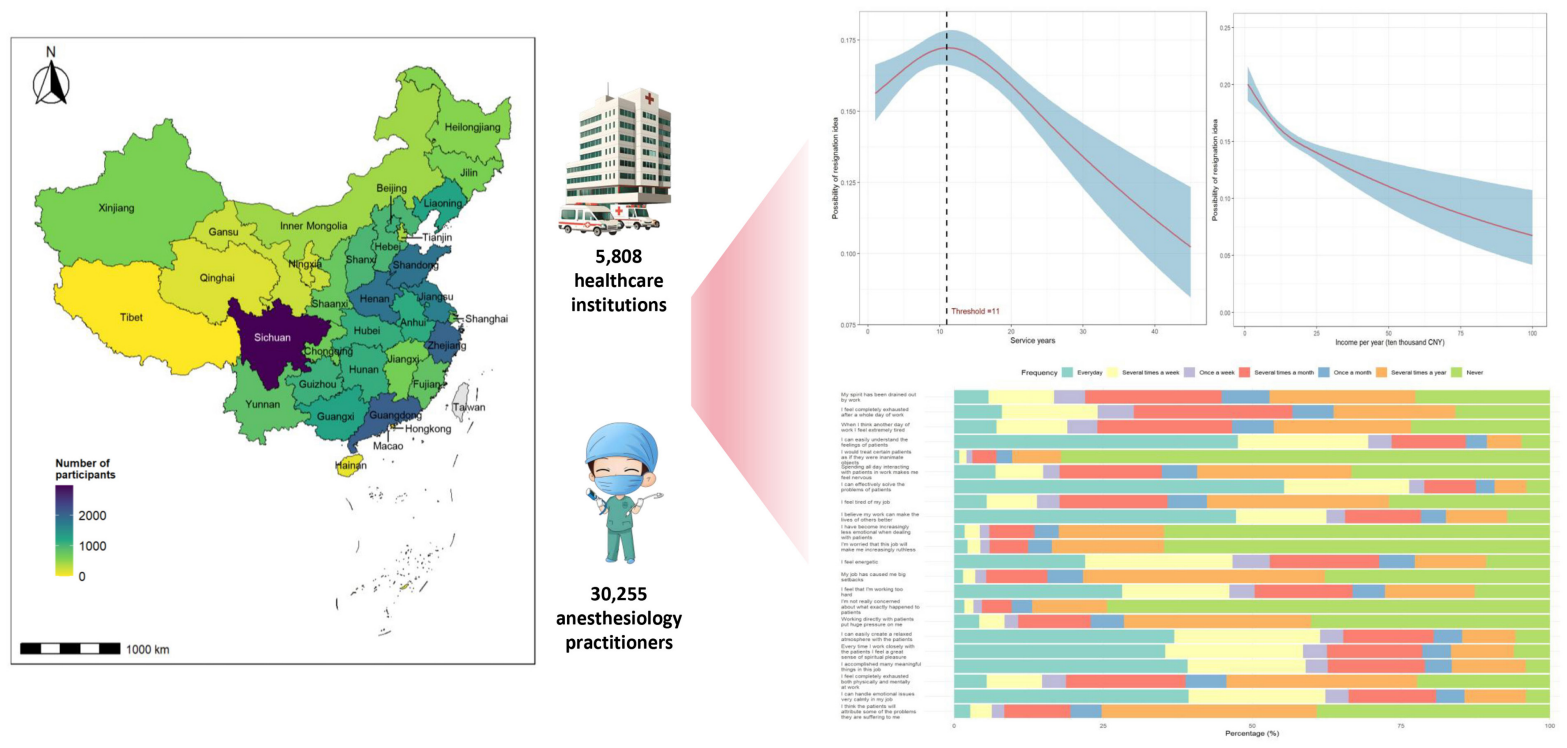
Anesthesiologists in China: a nationwide survey of working conditions, clinical practice, and career challenges. Despite demonstrating high clinical quality and professional fulfillment, Chinese anesthesiologists face substantial challenges including heavy workloads, occupational burnout, and career advancement barriers.

This nationwide cross-sectional survey, encompassing 30,255 anesthesiologists from 5,808 healthcare institutions across mainland China, provides a comprehensive portrayal of work condition, clinical practice, and career development of anesthesiologists in China (Figure 5). The findings reveal multifaceted challenges and offer valuable insights into this critical medical specialty.

The survey results underscore several key concerns. Notably, 16.3% of participants reported active resignation intention within the past month, a proportion that appears higher than rates reported in selected Western physician/anesthesiology samples (2, 23), although direct comparisons are limited by measurement differences. Furthermore, a significant proportion of anesthesiologists reported experiencing substantial occupational burnout and emotional exhaustion. Positive interventions such as optimized staffing, leadership support, schedule flexibility, and adjusted compensation are imperative to mitigate burnout and exhaustion within this workforce (24). Additionally, job satisfaction among anesthesiologists appears suboptimal, with 56.27% of participants reporting satisfaction or high satisfaction—a rate significantly lower than comparable cohorts in Western countries and relative to physicians overall (25). Thirdly, this survey highlights that the clinical practice of anesthesiologists confronts remarkable challenges, including medical disputes, violence exposure, and self-reported procedural errors which should receive adequate attention. Lastly, career advancement is impeded by barriers such as research publication requirements and professional title examination, with 62.8% of respondents identifying “research publication” as their principal barrier to promotion. This underscores the prevalence of a research-output-centric model for career progression in China. Collectively, these figures may exceed established global burnout benchmarks (26), suggesting distinct systemic pressures within Chinese anesthesiology.

Meanwhile, this study identified several noteworthy findings and modifiable associated factors. Contract workers exhibited a 43% higher risk of resignation compared to permanent staff, underscoring job insecurity as a significant driver. Economic factors, evidenced by the negative correlation with income and the impact of workload, demonstrated that each additional work hour or anesthesia case increased resignation likelihood. Conversely, protective factors included moderate exercise (2–3 sessions/week reduced risk by 49%, OR = 0.51) and marital stability with adult children (OR = 0.49). These findings reinforce evidence supporting resilience strategies and offer specific intervention targets (27–29). Furthermore, the inverted U-shaped relationship between service years and resignation risk, peaking at 11 years, reveals a critical mid-career vulnerability window. This extends current understanding by suggesting that accumulated systemic stressors (e.g., promotion barriers, plateauing compensation) converge at this career stage.

Previous studies on Chinese anesthesiologists have yielded valuable insights but were frequently constrained by modest sample sizes, regional biases, and limited scope. In contrast, our nationwide survey, using a larger and more geographically and institutionally diverse sample, identified a lower yet still alarmingly high burnout prevalence. Our study extends this evidence base by examining a comprehensive range of contributory factors and their interrelationships, thereby providing a more nuanced understanding of the challenges encountered by anesthesiologists across diverse hospital settings and regions nationwide.

There are about 105,732 anesthesiologists in China (16). The sample size of our study (*n* = 30,255) accounts for approximately 28.6% of the Chinese anesthesiologist workforce. To our knowledge, this study constitutes the largest and most comprehensive national survey of Chinese anesthesiologists' work conditions to date. Its innovation and strengths include: Firstly, the unprecedentedly large sample size and broad coverage enable the generalization of the findings to the national context, thereby providing a more comprehensive picture of the anesthesiologist workforce in China. Secondly, the multidimensional scope encompassing demographic and professional characteristics, work feelings and experiences, perceptions of clinical quality, job satisfaction, and career development challenges, facilitates a holistic analysis of the factors affecting practitioner sustainability. Thirdly, application of advanced statistical methodologies (e.g., restricted cubic spline and threshold effect analyses) allows for the elucidate of non-linear relationships between variables, revealing complex interaction dynamics. These strengths position our work as a significant advance in the literature, providing robust evidence to guide healthcare policy and clinical practice.

Despite its strengths, several limitations warrant acknowledgment. First, the cross-sectional design precludes the establishment of causal relationships between variables. This survey can only demonstrate associations rather than causal relationships between variables. Therefore, causal inferences should not be made from the present results. Longitudinal studies are warranted to further elucidate causal relationships. Second, the reliance on self-reported data introduces the potential for recall bias, social desirability bias, and subjective interpretation bias of the scales. Participants may not always provide completely accurate accounts of their experiences and perceptions. Third, while the survey covers a wide range of factors, unmeasured variables, such as individual personality traits, specific hospital culture, and local policy variations, may also influence the outcomes. Fourth, the unavoidable response bias caused by remote areas, the older population or extremely busy practitioners should be taken into consideration. Fifth, the study used voluntary non-probability sampling via the national professional network, which may lead to selection bias and limit the representativeness of the sample, although the sample size and coverage were the largest to date for this population. Future research employing systematic sampling strategies and addressing the limitations outlined above is warranted to further refine our understanding.

In general, this study provides a comprehensive national assessment of anesthesiologists' working conditions in China. While reporting intrinsic professional fulfillment, anesthesiologists face substantial challenges including excessive workloads, high burnout rates, prevalent resignation intentions, and significant career advancement barriers. These findings collectively indicate a workforce under considerable strain and necessitate urgent systemic interventions. Addressing these multifactorial issues is essential for cultivating a sustainable, high-quality anesthesia workforce and enhancing perioperative care delivery across China's healthcare system.

## 5 Implications for policy and practice

This large-scale national survey not only provides a comprehensive profile of working conditions among Chinese anesthesiologists but also identifies actionable targets for intervention. Based on the risk and protective factors identified, we propose a multi-level intervention framework as follows:

Institutional level: Implement mandatory limits on weekly working hours and daily anesthesia caseloads, enforce post-call rest periods, and deploy anesthesia nurse assistants to reduce non-clinical workload. Workplace wellness programs should promote sleep hygiene and regular physical activity, given their strong protective associations with reduced resignation intention.

Professional level: The title promotion system should be reformed to reduce reliance on research publications and formally recognize clinical excellence, teaching, and quality improvement. A distinct clinical promotion track should be established, particularly for anesthesiologists in primary and secondary hospitals.

Individual level: Mid-career anesthesiologists (approximately 5–15 service years) should be prioritized for career development support, including subspecialty training, leadership cultivation, and mentorship roles. Regular exercise and sleep quality monitoring should be integrated into occupational health protocols.

Societal level: National public awareness campaigns are urgently needed to improve recognition of anesthesiologists' contributions, thereby mitigating verbal violence and reinforcing professional dignity.

## 6 Conclusion

This study highlights critical challenges among anesthesiologists in China, including excessive workloads, high burnout, and career development barriers, despite high clinical efficacy and professional fulfillment. Targeted intervention strategy and evidence-based policy are urgently required to safeguard the sustainability of high-quality perioperative care in China.

## Data Availability

The raw data supporting the conclusions of this article will be made available by the authors, without undue reservation.
