# Supplementary material for: Anesthesiologists in China: a nationwide cross-sectional survey of working conditions, clinical practice, and career challenges
Source: Front Public Health. 2026 Jul 3;14:1846157. doi: 10.3389/fpubh.2026.1846157 (PMC13375972; doi:10.3389/fpubh.2026.1846157)
Supplement: Supplementary file 1 [file Data_Sheet_1.pdf]

Questionnaire Survey on the Current Working Situation of Anesthesiologists in China

Part One: Basic information

(This section contains 17 items)

1. **Your gender (compulsory)**
   ☐ Male ☐Female

2. **Your age____ (Only numbers are allowed, 18 - 99 years)**

3. **The full name of your hospital____**

4. **Your hospital level____**
   ☐Grade3A
   ☐Grade3
   ☐Grade2A
   ☐Grade2
   ☐Grade1
   ☐Ungraded
   ☐Other

5. **Your hospital type____**
   ☐Public general hospital
   ☐Public specialized hospital
   ☐Public TCM hospital
   ☐Private hospital
   ☐Others

6. **Your identity____**

☐Permanent staff

☐Contract staff

☐Rotational or trainees

☐Others

7. **Your profession____**

☐Certified doctor

☐ Physician assistant

☐Nurse

☐Technician

☐Other

8. **Your major____**

☐Anesthesiology

☐Clinical Medicine

☐Nursing

☐Others

9. **Your educational background____**

☐Associate degree

☐Bachelor's degree

☐Master's degree

☐Doctoral degree

☐Others

10. **Your profession title____**

☐Junior

☐Intermediate

☐Associate senior

☐Senior

☐Other

11. **The service years you have been engaged in anesthesia work____ (Only numbers are allowed, 0-70 years)**

12. **Your average week work hours____**

☐< 40 hours

☐40-50 hours

☐50-60 hours

☐60-70 hours

☐70-80 hours

☐>80 hours

13. The average number of anesthesia cases each day____

☐1 case

☐2 to 3 cases

☐4 to 5 cases

☐6 to 7 cases

☐8 to 10 cases

☐> 10 cases

14. Do you need to be on duty at the hospital____

☐Yes ☐No

15. The period of your on-duty at the hospital is____

☐≤3 days

☐4-5 days

☐6-7 days

☐8-10 days

☐>10 days

16. Your annual after-tax income is ____ten thousand CNY (Including all incomes such as salary, bonus, and subsidy, and only numbers are allowed)

17. Your marital status and family situation____

☐Unmarried

☐Married without children

☐Married with minor children

☐Married with adult children

☐Others

Part Two: Work feelings and experiences

The following sentences describe some feelings and situations that you might encounter in your daily work. Please read them carefully and, based on your actual situation, select the frequency that best suits you. (This section contains 22 items)

1. My spirit has been drained out by work.
☐Never
☐Several times a year
☐Once a month
☐Several times a month
☐Once a week
☐Several times a week
☐Everyday

2. I feel completely exhausted after a whole day of work.
☐Never
☐Several times a year
☐Once a month
☐Several times a month
☐Once a week
☐Several times a week
☐Everyday

3. When I woke up in the morning, thinking that I had to face another day of work, I felt extremely tired.
☐Never
☐Several times a year
☐Once a month
☐Several times a month
☐Once a week
☐Several times a week
☐Everyday

4. I can easily understand the feelings of patients.
☐Never
☐Several times a year
☐Once a month
☐Several times a month
☐Once a week

☐Several times a week
☐Everyday

5. I would treat certain patients as if they were inanimate objects without any feelings.
☐Never
☐Several times a year
☐Once a month
☐Several times a month
☐Once a week
☐Several times a week
☐Everyday

6. Spending the whole day interacting with people at work makes me feel extremely nervous.
☐Never
☐Several times a year
☐Once a month
☐Several times a month
☐Once a week
☐Several times a week
☐Everyday

7. I can effectively solve the problems of patients.
☐Never
☐Several times a year
☐Once a month
☐Several times a month
☐Once a week
☐Several times a week
☐Everyday

8. I feel tired of my job.
☐Never
☐Several times a year
☐Once a month
☐Several times a month
☐Once a week
☐Several times a week
☐Everyday

9. I believe my work can make the lives of others better.
☐Never
☐Several times a year

☐Once a month
☐Several times a month
☐Once a week
☐Several times a week
☐Everyday

10. Since taking up this job, I have become increasingly less emotional when dealing with patients.
☐Never
☐Several times a year
☐Once a month
☐Several times a month
☐Once a week
☐Several times a week
☐Everyday

11. I'm worried that this job will make me increasingly ruthless.
☐Never
☐Several times a year
☐Once a month
☐Several times a month
☐Once a week
☐Several times a week
☐Everyday

12. I feel energetic.
☐Never
☐Several times a year
☐Once a month
☐Several times a month
☐Once a week
☐Several times a week
☐Everyday

13. My job has caused me big setbacks.
☐Never
☐Several times a year
☐Once a month
☐Several times a month
☐Once a week
☐Several times a week
☐Everyday

14. I feel that I'm working too hard.

☐Never
☐Several times a year
☐Once a month
☐Several times a month
☐Once a week
☐Several times a week
☐Everyday

15. I'm not really concerned about what exactly happened to patients.
☐Never
☐Several times a year
☐Once a month
☐Several times a month
☐Once a week
☐Several times a week
☐Everyday

16. Working directly with patients has put huge pressure on me.
☐Never
☐Several times a year
☐Once a month
☐Several times a month
☐Once a week
☐Several times a week
☐Everyday

17. When I'm with the patients, I can easily create a relaxed atmosphere.
☐Never
☐Several times a year
☐Once a month
☐Several times a month
☐Once a week
☐Several times a week
☐Everyday

18. Every time I work closely with the patients, I feel a great sense of spiritual pleasure.
☐Never
☐Several times a year
☐Once a month
☐Several times a month
☐Once a week
☐Several times a week
☐Everyday

19.In this job, I accomplished many meaningful things.

☐Never
☐Several times a year
☐Once a month
☐Several times a month
☐Once a week
☐Several times a week
☐Everyday

20. At work, I feel completely exhausted both physically and mentally.

☐Never
☐Several times a year
☐Once a month
☐Several times a month
☐Once a week
☐Several times a week
☐Everyday

21. In my job, I can handle emotional issues very calmly.

☐Never
☐Several times a year
☐Once a month
☐Several times a month
☐Once a week
☐Several times a week
☐Everyday

22. I think the patients will attribute some of the problems they are suffering to me.

☐Never
☐Several times a year
☐Once a month
☐Several times a month
☐Once a week
☐Several times a week
☐Everyday

Part Three: Clinical quality and career development

(This section contains 21 items)

1.In general, are you satisfied with your job?
☐Extremely dissatisfied
☐Dissatisfied
☐Average satisfaction
☐Satisfied
☐Very satisfied

2.The frequency at which you encounter challenging cases was?
☐Few
☐Once a month
☐Once a week
☐Once every 2 to 3 days
☐Almost everyday

3. In the past three months, the average communication time you spent with each patient and their family members was?
Few
☐1to 3 minutes
☐4 to 5 minutes
☐6 to 10 minutes
☐>10 minutes

4. The frequency of your patients undergoing general anesthesia who were left in operating room for more than 5 minutes in past three months
☐None
☐Only once
☐Once or twice a month
☐Once or twice a week
☐Almost everyday

5. The frequency of you made mistakes in operations such as internal jugular puncture and catheterization, intraspinal anesthesia, etc. in past three months
☐None
☐Only once
☐Once or twice a month
☐Once or twice a week
☐Almost everyday

6.The frequency of you made medication errors such as incorrect drug type, dosage, concentration, route or administration speed in past three months

☐None
☐Only once
☐Once or twice a month
☐Once or twice a week
☐Almost everyday

7. The frequency of you made incorrect judgments regarding the condition of patients in past three months
☐None
☐Only once
☐Once or twice a month
☐Once or twice a week
☐Almost everyday

8.In the past three months, have you made any mistakes that caused harm to patients?
☐No
☐Yes

9. How many days of paid vacation do you have as a regular entitlement each year? ___(Only numbers are allowed, 0-366 days)

10. The biggest challenge you are facing in terms of professional title promotion is?
☐No difficulties
☐Clinical competence
☐Professional title examination
☐Paper publication
☐Competition among colleagues
☐Other difficulties

11. In the past year, have you ever experienced medical disputes?
☐No
☐Yes

12. In the past year, the frequency of verbal violence (using insulting, defamatory, contemptuous, mocking and other discriminatory and insulting language, but without any physical contact) that you have experienced from the patients was?
☐None
☐Less than once per month
☐Once per month
☐2 to 3 times per month
☐Once per week

☐2 to 5 times per week
☐Almost everyday

13. In the past year, the frequency of physical violence (which includes physical contact or violent acts using weapons) that you have experienced from the patients was?
☐None
☐Less than once per month
☐Once per month
☐2 to 3 times per month
☐Once per week
☐2 to 5 times per week
☐Almost everyday

14. In the past month, have you ever had the idea of resignation?
☐No
☐Yes

15. If you had the chance to choose again, what is the possibility that you would still choose current career?
☐Impossible
☐Less likely
☐Uncertain
☐More likely
☐Very likely

16. In the past month, your actual sleep duration each night was?
☐<5 hours
☐5 to 6 hours
☐6 to 7 hours
☐> 7 hours

17. In the past month, overall, how would you rate your sleep quality?
☐Very good
☐Good
☐Average
☐Poor
☐Very poor

18. In the past month, the frequency of your use of sleep aid medications was?
☐None
☐Less than once per week
☐1 to 2 times per week
☐≥3 times per week

19. Do you smoke? ("Smoking" is defined as having smoked more than 100 cigarettes cumulatively, while "quitting smoking" is defined as having completely stopped smoking for more than three months.)

☐No
☐Former smoker, but has quit
☐Yes, still smoking currently

20. Over the past year, how often have you consumed alcohol?

☐Never
☐Once or less per month
☐2 to 4 times per month
☐2 to 3 times per week
☐Four times or more per week

21. How many times do you exercise physically each week?

☐No exercise
☐Once a week
☐2 to 3 times a week
☐4 to 5 times a week
☐Almost everyday
