# Supplementary material for: Anesthesiologists in China: a nationwide cross-sectional survey of working conditions, clinical practice, and career challenges
Source: Front Public Health. 2026 Jul 3;14:1846157. doi: 10.3389/fpubh.2026.1846157 (PMC13375972; doi:10.3389/fpubh.2026.1846157)
Supplement: Supplementary file 2 [file Table_1.docx]

**Anesthesiologists in China: A nationwide cross-sectional survey of working conditions, clinical practice, and career** **challenges**

Yixun Lu^1†^, Changsheng Zhang^1†^, Weihua Wang^2^, Xuecai Lv^1^, Huikai Yang^1,3^, Yulong Ma^1^, Jiangbei Cao^1^, Yuguang Huang^4*^, Weidong Mi^1,5*^

**Supplementary** **material File 2**


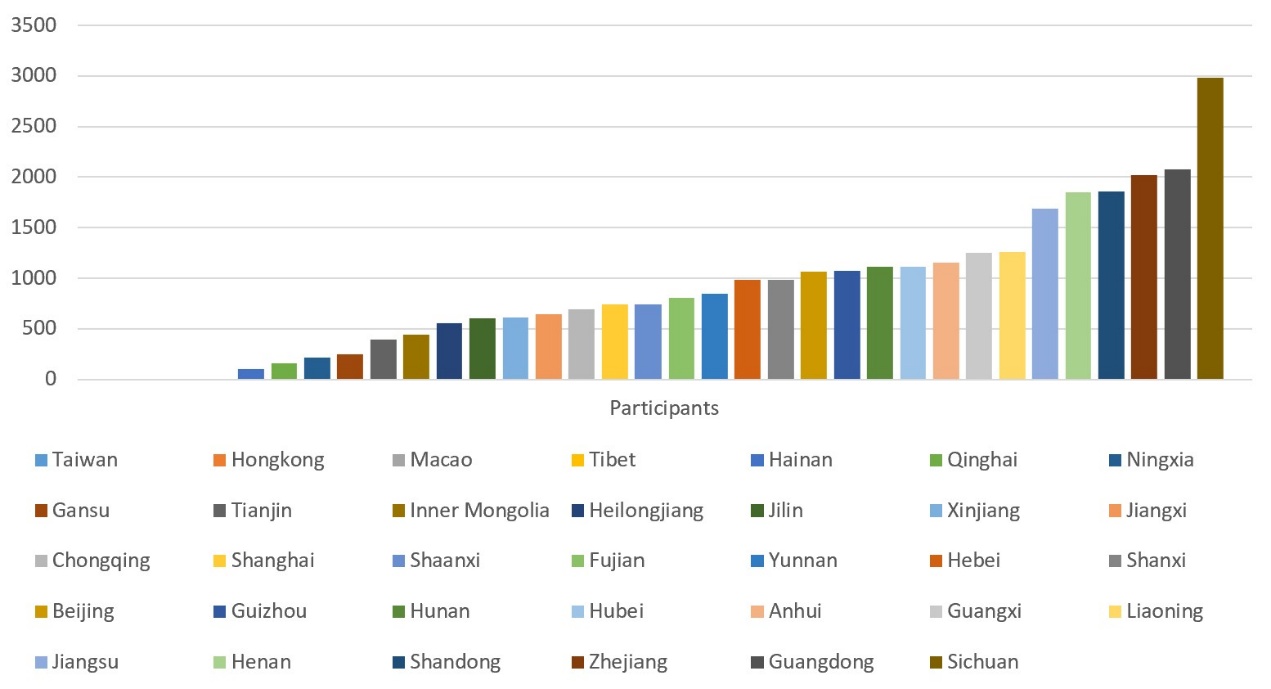


**Fig. S1** Distribution of participants in each province, city or autonomous region in China.

**Table S1.** Univariate analysis results of factors associated with resignation intention in participating anesthesiologists (N = 30,255).

| Characters | Odds ratio (95% CI) of resignation | P-value |
| --- | --- | --- |
| **Gender** |  |  |
| Male | 1.0 | Reference |
| Female | 0.91 (0.86, 0.97) | 0.0030 |
| **Age** | 0.98 (0.98, 0.99) | <0.0001 |
| **Hospital level** |  |  |
| Ungraded | 1.0 | Reference |
| Grade1 | 0.48 (0.24, 0.99) | 0.0453 |
| Grade2 | 0.93 (0.48, 1.78) | 0.8208 |
| Grade2A | 1.10 (0.58, 2.10) | 0.7648 |
| Grade3 | 1.20 (0.63, 2.29) | 0.5749 |
| Grade3A | 1.23 (0.65, 2.33) | 0.5258 |
| Others | 0.24 (0.09, 0.64) | 0.0046 |
| **Hospital type** |  |  |
| Private hospital | 1.0 | Reference |
| Public TCM hospital | 1.09 (0.92, 1.29) | 0.3451 |
| Public specialized hospital | 0.94 (0.82, 1.08) | 0.4200 |
| Public general hospital | 1.15 (1.04, 1.29) | 0.0096 |
| Other | 1.50 (0.96, 2.34) | 0.0740 |
| **Identity** |  |  |
| Permanent staff | 1.0 | Reference |
| Contract staff | 1.43 (1.34, 1.53) | <0.0001 |
| Rotational or trainees | 1.37 (1.04, 1.80) | 0.0237 |
| Others | 1.60 (1.19, 2.14) | 0.0016 |
| **Profession** |  |  |
| Certified doctor | 1.0 | Reference |
| Physician assistant | 0.67 (0.56, 0.81) | <0.0001 |
| Nurse | 0.60 (0.48, 0.75) | <0.0001 |
| Technician | 0.85 (0.49, 1.48) | 0.5743 |
| Others | 0.92 (0.62, 1.36) | 0.6866 |
| **Major** |  |  |
| Anesthesiology | 1.0 | Reference |
| Clinical Medicine | 1.09 (1.02, 1.17) | 0.0129 |
| Nursing | 0.61 (0.49, 0.77) | <0.0001 |
| Others | 1.12 (0.69, 1.81) | 0.6525 |
| **Education** |  |  |
| Doctoral degree | 1.0 | Reference |
| Master degree | 1.02 (0.84, 1.24) | 0.8447 |
| Bachelor degree | 1.03 (0.85, 1.24) | 0.7586 |
| Associate degree | 0.76 (0.61, 0.94) | 0.0135 |
| Others | 1.72 (0.34, 8.60) | 0.5109 |
| **Profession title** |  |  |
| Junior | 1.0 | Reference |
| Intermediate | 1.08 (1.00, 1.16) | 0.0365 |
| Associate senior | 1.04 (0.95, 1.14) | 0.3635 |
| Senior | 0.89 (0.76, 1.05) | 0.1747 |
| Other | 1.19 (0.90, 1.57) | 0.2230 |
| **Service years** | 0.99 (0.99, 1.00) | 0.0019 |
| **Week work hours** |  |  |
| <40 hours | 1.0 | Reference |
| 40 to 50 hours | 1.39 (1.18, 1.64) | <0.0001 |
| 50 to 60 hours | 1.96 (1.67, 2.31) | <0.0001 |
| 60 to 70 hours | 2.72 (2.29, 3.22) | <0.0001 |
| 70 to 80 hours | 2.89 (2.36, 3.53) | <0.0001 |
| >80 hours | 4.87 (3.95, 5.99) | <0.0001 |
| **Day anesthesia cases** |  |  |
| 1 case | 1.0 | Reference |
| 2 to 3 cases | 1.40 (1.21, 1.62) | <0.0001 |
| 4 to 5 cases | 1.98 (1.71, 2.29) | <0.0001 |
| 6 to 7 cases | 2.07 (1.76, 2.44) | <0.0001 |
| 8 to 10 cases | 1.99 (1.62, 2.44) | <0.0001 |
| >10 cases | 2.26 (1.82, 2.80) | <0.0001 |
| **On-call duty** |  |  |
| No | 1.0 | Reference |
| Yes | 1.66 (1.53, 1.79) | <0.0001 |
| **Period of on duty** |  |  |
| <=3 days | 1.0 | Reference |
| 4 to 5 days | 1.24 (1.11, 1.38) | 0.0001 |
| 6 to 7 days | 1.17 (1.05, 1.30) | 0.0058 |
| 8 to 10 days | 1.21 (1.07, 1.37) | 0.0019 |
| >10 days | 1.05 (0.90, 1.23) | 0.5131 |
| **Income per year** | 0.98 (0.98, 0.99) | <0.0001 |
| **Marital status** |  |  |
| Unmarried | 1.0 | Reference |
| Married without children | 0.88 (0.78, 1.00) | 0.0469 |
| Married with minor children | 0.90 (0.83, 0.97) | 0.0095 |
| Married with adult children | 0.49 (0.43, 0.57) | <0.0001 |
| Others | 1.24 (0.89, 1.74) | 0.2038 |
| **Sleep quality** |  |  |
| Very good | 1.0 | Reference |
| Good | 1.49 (1.28, 1.75) | <0.0001 |
| Average | 2.90 (2.51, 3.36) | <0.0001 |
| Poor | 6.22 (5.35, 7.23) | <0.0001 |
| Very poor | 12.88 (10.79, 15.38) | <0.0001 |
| **Smoking status** |  |  |
| No | 1.0 | Reference |
| Former smoker, but has quit | 0.98 (0.86, 1.12) | 0.7779 |
| Yes, still smoking currently | 1.60 (1.47, 1.75) | <0.0001 |
| **Alcohol status** |  |  |
| Never | 1.0 | Reference |
| Once or less per month | 1.59 (1.49, 1.71) | <0.0001 |
| 2 to 4 times per month | 1.75 (1.60, 1.92) | <0.0001 |
| 2 to 3 times per week | 2.18 (1.87, 2.53) | <0.0001 |
| Four times or more per week | 2.74 (2.09, 3.60) | <0.0001 |
| **Exercise frequency** |  |  |
| No exercise | 1.0 | Reference |
| Once a week | 0.57 (0.53, 0.62) | <0.0001 |
| 2 to 3 times a week | 0.51 (0.46, 0.55) | <0.0001 |
| 4 to 5 times a week | 0.61 (0.51, 0.72) | <0.0001 |
| Almost everyday | 0.57 (0.48, 0.69) | <0.0001 |

**Table S2.** Multivariable logistic regression analysis of factors independently associated with resignation intention among participating anesthesiologists (N = 30,255).

| **Variable** | **Variable** | | **OR** | **P-adjust** |
| --- | --- | --- | --- | --- |
|  | **details** | **(95% CI)** | | **(Bonferroni)** |
| **Gender (vs Male)** |  |  | |  |
|  | Female | 1.08(0.96-1.21) | | 1 |
| **Hospital level (vs Ungraded)** |  |  | |  |
|  | Grade 1 | 0.52(0.17-1.57) | | 1 |
|  | Grade 2 | 0.73(0.26-2.03) | | 1 |
|  | Grade 2A | 0.78(0.28-2.14) | | 1 |
|  | Grade 3 | 0.8(0.29-2.22) | | 1 |
|  | Grade 3A | 0.82(0.29-2.26) | | 1 |
|  | Others | 0.29(0.06-1.31) | | 0.719 |
| **Service years** | Service years | 1.03(1.02-1.05) | | <0.001 |
| **Week work hours (vs <40 hours)** |  |  | |  |
|  | 40 to 50 hours | 1.09(0.84-1.41) | | 1 |
|  | 50 to 60 hours | 1.31(1.01-1.69) | | 0.107 |
|  | 60 to 70 hours | 1.59(1.21-2.11) | | <0.001 |
|  | 70 to 80 hours | 1.59(1.15-2.19) | | 0.001 |
|  | >80 hours | 2.39(1.71-3.33) | | <0.001 |
| **Day anesthesia cases (vs 1 case)** |  |  | |  |
|  | 2 to 3 cases | 0.93(0.74-1.18) | | 1 |
|  | 4 to 5 cases | 1.1(0.86-1.39) | | 1 |
|  | 6 to 7 cases | 1.11(0.84-1.45) | | 1 |
|  | 8 to 10 cases | 1.08(0.77-1.52) | | 1 |
|  | >10 cases | 1.29(0.91-1.83) | | 1 |
| **Need on-call duty (vs No)** |  |  | |  |
|  | Yes | 1.21(1.05-1.38) | | 0.002 |
| **Income per year** | Income per year | 0.98(0.97-0.99) | | <0.001 |
| **Marital status (vs Unmarried)** |  |  | |  |
|  | Married without children | 0.97(0.8-1.18) | | 1 |
|  | Married with minor children | 0.81(0.69-0.94) | | 0.001 |
|  | Married with adult children | 0.52(0.39-0.69) | | <0.001 |
|  | Others | 1.04(0.6-1.79) | | 1 |
| **Sleep quality (vs Very poor)** |  |  | |  |
|  | Poor | 0.53(0.43-0.64) | | <0.001 |
|  | Average | 0.27(0.22-0.33) | | <0.001 |
|  | Good | 0.16(0.13-0.19) | | <0.001 |
|  | Very good | 0.12(0.09-0.16) | | <0.001 |
| **Smoking (vs No)** |  |  | |  |
|  | Former smoker, but has quit | 0.94(0.76-1.17) | | 1 |
|  | Yes, still smoking currently | 1.14(0.98-1.34) | | 0.583 |
| **Alcohol (vs Never)** |  |  | |  |
|  | Once or less per month | 1.49(1.33-1.68) | | <0.001 |
|  | 2 to 4 times per month | 1.69(1.45-1.99) | | <0.001 |
|  | 2 to 3 times per week | 2.01(1.56-2.59) | | <0.001 |
|  | Four times or more per week | 2.41(1.55-3.75) | | <0.001 |
| **Exercise (vs No exercise)** |  |  | |  |
|  | Once a week | 0.67(0.59-0.75) | | <0.001 |
|  | 2 to 3 times a week | 0.64(0.56-0.74) | | <0.001 |
|  | 4 to 5 times a week | 0.8(0.61-1.06) | | 0.873 |
|  | Almost everyday | 0.76(0.57-1.02) | | 0.269 |

OR, odds ratio; CI, confidence interval.

**Table S3.** Full results of self-reported work feelings and occupational experiences of anesthesiologists in China (N = 30,255).

| **Questionnaire items** | **Statistics [N (%)]** |
| --- | --- |
| **My spirit has been drained out by work.** |  |
| Never | 6829 (22.57%) |
| Several times a year | 7419 (24.52%) |
| Once a month | 2423 (8.01%) |
| Several times a month | 6942 (22.94%) |
| Once a week | 1574 (5.20%) |
| Several times a week | 3327 (11.00%) |
| Everyday | 1741 (5.75%) |
| **I feel completely exhausted after a whole day of work.** |  |
| Never | 4813 (15.91%) |
| Several times a year | 6173 (20.40%) |
| Once a month | 2091 (6.91%) |
| Several times a month | 8064 (26.65%) |
| Once a week | 1842 (6.09%) |
| Several times a week | 4864 (16.08%) |
| Everyday | 2408 (7.96%) |
| **When I woke up in the morning, thinking that I had to face another day of work, I felt extremely tired.** |  |
| Never | 7057 (23.33%) |
| Several times a year | 6970 (23.04%) |
| Once a month | 2129 (7.04%) |
| Several times a month | 6830 (22.57%) |
| Once a week | 1537 (5.08%) |
| Several times a week | 3594 (11.88%) |
| Everyday | 2138 (7.07%) |
| **I can easily understand the feelings of patients.** |  |
| Never | 1436 (4.75%) |
| Several times a year | 1767 (5.84%) |
| Once a month | 1068 (3.53%) |
| Several times a month | 3761 (12.43%) |
| Once a week | 1196 (3.95%) |
| Several times a week | 6621 (21.88%) |
| Everyday | 14406 (47.62%) |
| **I would treat certain patients as if they were inanimate objects without any feelings.** |  |
| Never | 24844 (82.12%) |
| Several times a year | 2469 (8.16%) |
| Once a month | 806 (2.66%) |
| Several times a month | 1228 (4.06%) |
| Once a week | 290 (0.96%) |
| Several times a week | 372 (1.23%) |
| Everyday | 246 (0.81%) |
| **Spending the whole day interacting with people at work makes me feel extremely nervous.** |  |
| Never | 10077 (33.31%) |
| Several times a year | 7837 (25.90%) |
| Once a month | 1807 (5.97%) |
| Several times a month | 5181 (17.12%) |
| Once a week | 838 (2.77%) |
| Several times a week | 2429 (8.03%) |
| Everyday | 2086 (6.89%) |
| **I can effectively solve the problems of patients.** |  |
| Never | 1188 (3.93%) |
| Several times a year | 1626 (5.37%) |
| Once a month | 955 (3.16%) |
| Several times a month | 2612 (8.63%) |
| Once a week | 773 (2.55%) |
| Several times a week | 6350 (20.99%) |
| Everyday | 16751 (55.37%) |
| **I feel tired of my job.** |  |
| Never | 8178 (27.03%) |
| Several times a year | 9241 (30.54%) |
| Once a month | 2008 (6.64%) |
| Several times a month | 5476 (18.10%) |
| Once a week | 1156 (3.82%) |
| Several times a week | 2560 (8.46%) |
| Everyday | 1636 (5.41%) |
| **I believe my work can make the lives of others better.** |  |
| Never | 2168 (7.17%) |
| Several times a year | 3108 (10.27%) |
| Once a month | 1277 (4.22%) |
| Several times a month | 3845 (12.71%) |
| Once a week | 958 (3.17%) |
| Several times a week | 4588 (15.16%) |
| Everyday | 14311 (47.30%) |
| **Since taking up this job, I have become increasingly less emotional when dealing with patients.** |  |
| Never | 19598 (64.78%) |
| Several times a year | 5355 (17.70%) |
| Once a month | 1242 (4.11%) |
| Several times a month | 2285 (7.55%) |
| Once a week | 482 (1.59%) |
| Several times a week | 770 (2.55%) |
| Everyday | 523 (1.73%) |
| **I'm worried that this job will make me increasingly ruthless.** |  |
| Never | 19596 (64.77%) |
| Several times a year | 5701 (18.84%) |
| Once a month | 1212 (4.01%) |
| Several times a month | 1953 (6.46%) |
| Once a week | 475 (1.57%) |
| Several times a week | 651 (2.15%) |
| Everyday | 667 (2.20%) |
| **I feel energetic.** |  |
| Never | 3235 (10.69%) |
| Several times a year | 3631 (12.00%) |
| Once a month | 1802 (5.96%) |
| Several times a month | 5566 (18.40%) |
| Once a week | 1888 (6.24%) |
| Several times a week | 7491 (24.76%) |
| Everyday | 6642 (21.95%) |
| **My job has caused me big setbacks.** |  |
| Never | 11433 (37.79%) |
| Several times a year | 12287(40.61%) |
| Once a month | 1808 (5.98%) |
| Several times a month | 3105 (10.26%) |
| Once a week | 561 (1.85%) |
| Several times a week | 616 (2.04%) |
| Everyday | 445 (1.47%) |
| **I feel that I'm working too hard.** |  |
| Never | 3802 (12.57%) |
| Several times a year | 4575 (15.12%) |
| Once a month | 1655 (5.47%) |
| Several times a month | 4962 (16.40%) |
| Once a week | 1286 (4.25%) |
| Several times a week | 5445 (18.00%) |
| Everyday | 8530 (28.19%) |
| **I'm not really concerned about what exactly happened to patients.** |  |
| Never | 22504 (74.38%) |
| Several times a year | 3798 (12.55%) |
| Once a month | 1040 (3.44%) |
| Several times a month | 1514 (5.00%) |
| Once a week | 437 (1.44%) |
| Several times a week | 453 (1.50%) |
| Everyday | 509 (1.68%) |
| **Working directly with patients has put huge pressure on me.** |  |
| Never | 12134 (40.11%) |
| Several times a year | 9500 (31.40%) |
| Once a month | 1692 (5.59%) |
| Several times a month | 3688 (12.19%) |
| Once a week | 701 (2.32%) |
| Several times a week | 1282 (4.24%) |
| Everyday | 1258 (4.16%) |
| **When I'm with the patients, I can easily create a relaxed atmosphere.** |  |
| Never | 1739 (5.75%) |
| Several times a year | 2709 (8.95%) |
| Once a month | 1456 (4.81%) |
| Several times a month | 4590 (15.17%) |
| Once a week | 1181 (3.90%) |
| Several times a week | 7407 (24.48%) |
| Everyday | 11173 (36.93%) |
| **Every time I work closely with the patients, I feel a great sense of spiritual pleasure.** |  |
| Never | 1830 (6.05%) |
| Several times a year | 3189 (10.54%) |
| Once a month | 1463 (4.84%) |
| Several times a month | 4838 (15.99%) |
| Once a week | 1226 (4.05%) |
| Several times a week | 6993 (23.11%) |
| Everyday | 10716 (35.42%) |
| **In this job, I accomplished many meaningful things.** |  |
| Never | 1223 (4.04%) |
| Several times a year | 3761 (12.43%) |
| Once a month | 1366 (4.51%) |
| Several times a month | 4930 (16.29%) |
| Once a week | 1124 (3.72%) |
| Several times a week | 6003 (19.84%) |
| Everyday | 11848 (39.16%) |
| **At work, I feel completely exhausted both physically and mentally.** |  |
| Never | 6759 (22.34%) |
| Several times a year | 9668 (31.96%) |
| Once a month | 2092 (6.91%) |
| Several times a month | 6068 (20.06%) |
| Once a week | 1211 (4.00%) |
| Several times a week | 2809 (9.28%) |
| Everyday | 1648 (5.45%) |
| **In my job, I can handle emotional issues very calmly.** |  |
| Never | 1209 (4.00%) |
| Several times a year | 3131 (10.35%) |
| Once a month | 1440 (4.76%) |
| Several times a month | 4444 (14.69%) |
| Once a week | 1183 (3.91%) |
| Several times a week | 6944 (22.95%) |
| Everyday | 11904 (39.35%) |
| **I think the patients will attribute some of the problems they are suffering to me.** |  |
| Never | 11854 (39.18%) |
| Several times a year | 10919 (36.09%) |
| Once a month | 1580 (5.22%) |
| Several times a month | 3363 (11.12%) |
| Once a week | 644 (2.13%) |
| Several times a week | 1084 (3.58%) |
| Everyday | 811 (2.68%) |
